# Clinical Insights Into Heritable Cardiomyopathies

**DOI:** 10.3389/fgene.2021.663450

**Published:** 2021-04-28

**Authors:** Hugo R. Martinez, Gary S. Beasley, Noah Miller, Jason F. Goldberg, John L. Jefferies

**Affiliations:** ^1^The Heart Institute, Le Bonheur Children’s Hospital, The University of Tennessee Health Science Center, Memphis, TN, United States; ^2^The Cardiovascular Institute, The University of Tennessee Health Science Center, Memphis, TN, United States

**Keywords:** genetic expression, heritable cardiomyopathies, cardiac phenotypes, genetic testing, myocardial disease

## Abstract

Cardiomyopathies (CMs) encompass a heterogeneous group of structural and functional abnormalities of the myocardium. The phenotypic characteristics of these myocardial diseases range from silent to symptomatic heart failure, to sudden cardiac death due to malignant tachycardias. These diseases represent a leading cause of cardiovascular morbidity, cardiac transplantation, and death. Since the discovery of the first locus associated with hypertrophic cardiomyopathy 30 years ago, multiple loci and molecular mechanisms have been associated with these cardiomyopathy phenotypes. Conversely, the disparity between the ever-growing landscape of cardiovascular genetics and the lack of awareness in this field noticeably demonstrates the necessity to update training curricula and educational pathways. This review summarizes the current understanding of heritable CMs, including the most common pathogenic gene variants associated with the morpho-functional types of cardiomyopathies: dilated, hypertrophic, arrhythmogenic, non-compaction, and restrictive. Increased understanding of the genetic/phenotypic associations of these heritable diseases would facilitate risk stratification to leveraging appropriate surveillance and management, and it would additionally provide identification of family members at risk of avoidable cardiovascular morbidity and mortality.

## Introduction

Cardiomyopathies (CMs) are a group of disorders in which the muscle of the heart (myocardium) becomes structurally and functionally abnormal, leading to systolic dysfunction, diastolic dysfunction, and/or tachyarrhythmias (Arbustini et al., 2013). The global burden of genetically driven cardiomyopathies is difficult to quantify given the limited epidemiological studies representing a global framework. However, just alone, hypertrophic cardiomyopathy is considered the most common inherited cardiovascular disease, estimated to be present in 1:200 adult individuals. This information was generated after taking into account clinical profiles based on advanced imaging and familial transmission rates (Maron et al., 2018). Similarly, the prevalence of dilated cardiomyopathy has been estimated in the range of 1:250 adults (Hershberger et al., 2013). Furthermore, a recent meta-analysis incorporating more than 20 studies found strong prevalence in first-degree family members of the probands (up to 65%), which displays evidence of the high degree of heritability in these disorders (Petretta et al., 2011; Watkins et al., 2011; Ware, 2015). In the pediatric population, a cardiomyopathy registry concluded that the prevalence of heart failure amongst children with cardiomyopathy is relatively elevated, representing a leading cause of morbidity and mortality for this aged group (Wilkinson et al., 2010; McKenna et al., 2017).

Cardiomyopathies have been incorporated into multiple classifications over the past decades ranging from modest to more sophisticated schemes based on the genetic origin of the disease; in fact, the MOGES nosology incorporates a morpho-functional

phenotype (M), organ(s) involved (O), the genetic inheritance pattern (G), an etiological annotation (E) including genetic defects or underlying disease/substrates, and the functional status (S) of a particular patient based on heart failure symptoms (Maron et al., 2006; Elliott et al., 2008; Arbustini et al., 2013). However, classification systems are limited, due to the genetic and phenotypic heterogeneity expressed in myocardial diseases, as well as the limitations of a particular classification system to satisfy multiple scientific and clinical disciplines.

The common modes of inheritance for these disorders include autosomal dominant, autosomal recessive, X-linked and mitochondrial, but complex modes of Mendelian inheritance, mosaicisms, variable expressivity, and incomplete penetrance have also been described (Charron, 2006).

From the molecular point of view, CMs may originate from a pathogenic variation manifesting the disease (single-gene disorder), multiple pathogenic variations in the same gene (compound heterozygosity), or from a combination of variations in different genes (digenic heterozygosity) (Towbin, 2014; Li et al., 2019). Moreover, the incorporation of advanced DNA and RNA sequencing, has led to a comprehensive assessment of modifiers at other loci, gene promoters, loci enhancers, copy number variants (deletion/duplication) and molecular regulatory regions associated with “classic” phenotypes of cardiomyopathy, more severe phenotypic expressions, overlapping sub-types, and the co-occurrence of myocardial disease and tachyarrhythmias (Weischenfeldt et al., 2013; Towbin, 2014; Li et al., 2019; Lipshultz et al., 2019; Minoche et al., 2019). In this review, we focus on common genetic associations with the classic phenotypes of heritable cardiomyopathies, such as: dilated cardiomyopathy (DCM), hypertrophic cardiomyopathy (HCM), arrhythmogenic cardiomyopathy (ACM), non-compaction cardiomyopathy (LVNC), and restrictive cardiomyopathy (RCM); providing an opportunity to guide clinical management based upon known genetic underpinnings.

## Material Content

### Dilated Cardiomyopathy

**FIGURE 1:**
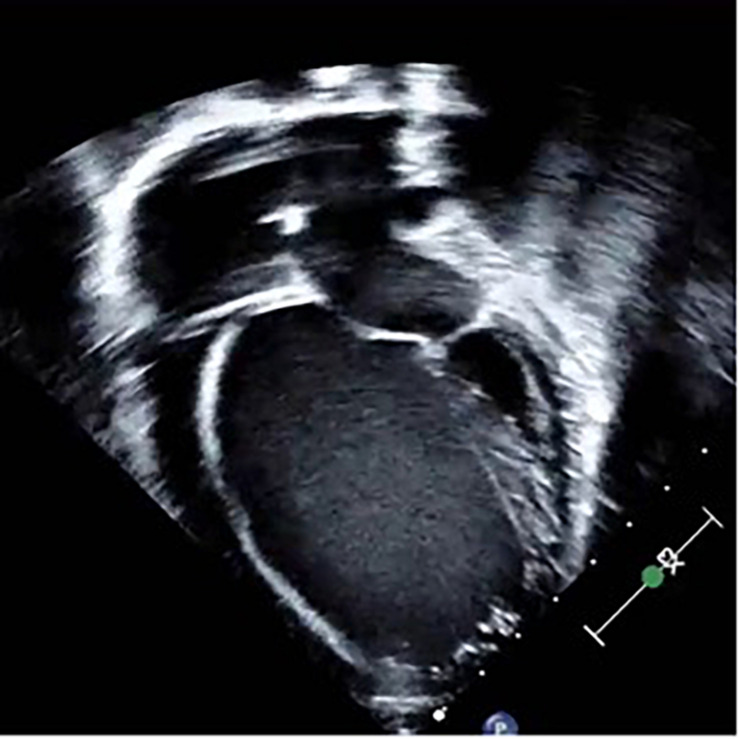
Two-dimensional, apical 4-chamber echocardiographic image depicting an enlarged ventricle with spherical geometry and bilateral atrial enlargement in a patient harboring dilated cardiomyopathy secondary to a pathogenic gene variant in the *TTN* gene.

Dilated cardiomyopathy is typically characterized by eccentric ventricular remodeling and decreased systolic function; and it can be detected in asymptomatic individuals, in those with heart failure symptoms, or in association with arrhythmias (Jefferies and Towbin, 2010) (Figure 1). Globally, DCM is one of the most common forms of cardiomyopathy, and it represents a leading cause of cardiac transplantation in children and adults (Lipshultz et al., 2019). The etiology of DCM can be broadly categorized into genetic, acquired, or mixed. Unfortunately, in many cases, no etiology can be found, and the disease is deemed idiopathic. Of interest, it is estimated that between 25 and 60% of patients with idiopathic DCM harbor positive family history for the disease, suggesting an underlying genetic predisposition (Petretta et al., 2011). Most genetically triggered cases of DCM are transmitted in an autosomal dominant pattern exhibiting variable penetrance. Other forms of inheritance include autosomal recessive, X-linked, and mitochondrial, have been described more frequent in the pediatric population (Arbustini et al., 2013).

Familial DCM occurs frequently given the high heritability of this disorder, but this approximation is subject to change as a more critical evaluation of the genes linked to DCM continues to evolve and certain gene variations shift to be certified as non-pathogenic (Petretta et al., 2011).

A conventional classification of DCM has been categorized based on the presence or absence of systemic disease. Thus, dividing DCM into syndromic and non-syndromic forms is a practical approach to evaluating this highly heterogeneous disease (Table 1). The diagnostic rate for gene testing in non-syndromic DCM is about 46–73% in tertiary centers with cardiovascular genetic expertise (Tayal et al., 2017) but it might be confounded by insufficient control for population variation. Over the past decade, over 100 genes in total have been linked to DCM in the Human Gene Mutation Database and the Online Mendelian Inheritance in Man (Fatkin et al., 2019; Online Mendelian Inheritance in Man, 2019) (Table 2). From these genes, a large-scale analysis revealed that truncating variants in the titin gene (*TNN*) are the most common disease-causative mutations in non-syndromic DCM (McNally et al., 2013; Nouhravesh et al., 2016). Other core genes include *MYH7*, *TNNT2* (encoding troponin T2) and *TPM1* (encoding tropomyosin 1). *LMNA* (encoding a nuclear envelope protein, laming A/C), has been associated with the arrhythmogenic type of DCM, as well and other skeletal myopathies such as Emery-Dreifuss, limb-girdle, and LMNA-related congenital muscular dystrophy (Maggi et al., 2016). Other rare pathogenic variants (minor allele frequency) implicated in non-syndromic DCM include genes coding for the sarcomere and Z-disk (actin, myosin-binding protein C3, myopalladin, nebulette, ZASP), cytoskeleton (dystrophin, desmin), nuclear envelope (emerin), mitochondria (tafazzin), sarcoplasmic reticulum, desmosomes, ion channels, and transcription factors (Martinez et al., 2014; McNally and Mestroni, 2017; Tayal et al., 2017; Hershberger et al., 2018a). X-linked cardiomyopathy has been reported as an isolated disease of the heart or associated with skeletal myopathies such as Duchenne and Becker muscular dystrophy (Towbin and Ortiz-Lopez, 1994; Davies and Nowak, 2006; Puckelwartz and McNally, 2011). It is worth mentioning that female carriers of X-linked dystrophinopathies with skewed inactivation of the healthy X chromosome, may present illness manifested by muscle weakness, myocardial dysfunction and heart failure. Depending on the degree of functional loss of the healthy X chromosome, female carriers may present symptoms as early as during the first decade of life (Martinez et al., 2011). Thus, starting phenotype screenings of female carriers of X-linked dystrophinopathies during childhood may be warranted.

**TABLE 1:** Typical features of common types of syndromic dilated cardiomyopathy.

Disease	Pathogenic gene variant and protein product	Patterns of inheritance	Clinical features
Barth syndrome	*TAZ/G4.5* – Tafazzin	XL	Male infants with cardioskeletal myopathy. Endocardial fibroelastosis; LVNC or HCM may be seen. Neutropenia, growth delays. 3-methylglutaconic aciduria (blood/urine)
Carvajal syndrome	*DSP* – Desmoplakin	AR	Brittle scalp with wooly hairs. Palmoplantar keratoderma.
Duchenne and Becker muscular dystrophy	*DMD* – Dystrophin	XL	Male children with muscle weakness. Pseudohypertrophy of calf muscles. Gower maneuver. Increased serum creatine kinase. Becker’s present later. Female carriers are variably affected.
Emery-dreifuss muscular dystrophy	*LMNA* – Lamin A/C *EMD* – Emerin *FHL1* – Four and a half LIM Domain 1	*EMD* and *FHL1: XL LMNA*: AD, AR	Childhood-onset muscle weakness. Joint contractures. Heart block and arrhythmias (by the second decade of life).
Laing distal myopathy	*MYH7* – ß-myosin heavy chain	AD	Initial weakness of the toes and fingers. A slow progression to central muscles. Tremors. Variable presentation from childhood to decades.
Limb-girdle muscular dystrophy 1B	*LMNA* – Lamin A/C	AD, AR	Affects voluntary proximal skeletal muscles. Presentation later in childhood to adulthood. Arrhythmias.

*AD, autosomal dominant; AR, autosomal recessive; XL, X-linked.*

**TABLE 2:** List of common genes and patterns of inheritance associated with DCM.

Gene	Protein	Mode of inheritance	Cardiac phenotype	OMIM#	Locus
*ABCC9*	ATP-binding cassette	AD	DCM	601439	14q12-q22
*ACTC1*	Actin-alpha cardiac muscle	AD	DCM, LVNC, ACM, HCM	102540	5q31.1
*ACTN2*	Actinin alpha-2	AD	DCM, HCM	102573	6q22.1
*AKAP9*	A-kinase anchor protein 9	AD	DCM	604001	2q32.1-q32.3
*ALMS1*	Centrosome and basal body associated protein	AR	DCM	606844	10p14-p12
*ALPK3*	Alpha kinase 3	AR	DCM, HCM	617608	1p36.32
*ANKRD1*	Ankyrin repeat domain-containing protein 1	AD	DCM, HCM	609599	7q36.1
*BAG3*	Bcl2-associated athanogene 3	AD	DCM, RCM, HCM	603883	14q24.3
*CASQ2*	Calsequestrin 2	AR, AD	DCM, LVNC	114251	6q22.31
*CAV3*	Caveolin 3	AD	DCM, HCM	601253	12q24.13
*CRYAB*	Crystallin	AD	DCM	123590	11q23.1
*CSRP3*	Cysteine and glycinin Protein 3	AD	DCM, HCM	600824	12p12.1
*CTF1*	Cardiotrophin 1	AR, AD	DCM	600435	1p13.1
*DES*	Desmin	AD, AR	DCM, ACM, RCM	125660	17q21
*DMD*	Dystrophin	XL	DCM	300377	3p25.3
*DOLK*	Dolichol kinase	AR	DCM	610746	7q33
*DSC2*	Desmocollin 2	AD, AR	DCM, ACM	600271	Xq22
*DSG2*	Desmoglein 2	AD	DCM, ACM	125671	15q24.1
*DSP*	Desmoplakin	AD, AR	DCM, ACM	125485	11p15.5
*DTNA*	Dystrobrevin alpha	AD	DCM, LVNC	601239	2q31
*EMD*	Emerin	XL	DCM	300384	11q23.1
*EYA4*	Eyes absent Drosophila homolog 4	AD	DCM	603550	11p15.1
*FHL1*	Four and a half LIM domain 1	XL	DCM, HCM	300163	15q22.31
*FKRP*	Fukutin-related protein	AR	DCM	606596	10q21.3
*FKTN*	Fukutin	AR	DCM	607440	2q35
*FLNC*	Filamin C	AD	DMC, RCM, HCM, ACM	102565	10q22.2
*GATA4*	Gata-binding protein 4	AD	DCM	600576	Xq21.2-p21.1
*GATAD1*	Gata zinc finger domain-containing protein 1	AR	DCM	614518	9q34.11
*GLA*	Galactosidase alpha	XL	DCM, HCM	300644	18q11.2
*ILK*	Integrin-linked kinase	AD	DCM	602366	18q12.1
*JUP*	Junction plakoglobin	AD, AR	DCM, ACM	173325	2p13.1
*LAMA4*	Laminin alpha-4	AD	DCM	600133	18q12.1
*LAMP2*	Lysosome-associated membrane protein 2	XL	DCM, HCM	309060	3p25.2
*LDB3*	Lim domain binding 3	AD	DCM, LVNC, ACM, HCM	605906	2p22.1
*LMNA*	Lamin A/C	AD, AR	DCM, LVNC, ACM, HCM	150330	1q22
*LRRC10*	Leucine-rich repeat-containing Protein 10	AD, AR	DCM	610846	4q21.3
*MYBPC3*	Myosin binding protein C	AD	DCM, LVNC, RCM, HCM	600958	Xq28
*MYH6*	Myosin heavy chain 6	AD	DCM, HCM	160710	10q25.2
*MYH7*	Myosin heavy chain 7	AD	DCM, LVNC, RCM, HCM	160760	7p14.2
*MYL2*	Myosin light chain 2	AD	DCM, HCM	160781	3p21.3-p21.2
*MYL3*	Myosin light chain 3	AD, AR	DCM, HCM, RCM	160790	1q32
*MYLK2*	Myosin light chain kinase 2	AD	DCM, HCM	606566	20q13.31
*MYOT*	Myotilin	AD	DCM	604103	Xq28
*MYOZ2*	Myozenin 2	AD	DCM, RCM, HCM	605602	3p25.1
*MYPN*	Myopalladin	AD	DCM, RCM, HCM	608517	12q23.1
*NEBL*	Nebulette	AD	DCM	605491	6q23.2
*NEXN*	Nexilin	AD	DCM, HCM	613121	1q22
*NKX2-5*	Nk2 homeobox 5	AD	DCM	600584	Xq26.3
*PDLIM3*	Pdz and lim domain protein 3	AD	DCM, HCM	605899	1q43
*PKP2*	Plakophilin 2	AD	DCM, ACM	602861	11p15.4
*PLN*	Phospholamban	AD	DCM, ACM, HCM	172405	4q12
*PRDM16*	PR domain-containing protein 16	AD	DCM, LVNC	605557	6q21
*PRKAG2*	Protein kinase amp-activated non-catalytic	AD	DCM, HCM	602743	4q26-q27
*RBM20*	RNA-binding motif protein 20	AD	DCM	613171	2q12.2
*RYR2*	Ryanodine receptor 2	AD	DCM, HCM, ACM	180902	12p11
*SCN5A*	Sodium channel voltage-gated	AD	DCM, ACM	600163	20q13.12
*SGCA*	Sarcoglycan alpha	AR	DCM	600119	1q25.2
*SGCB*	Sarcoglycan beta	AR	DCM	600900	15q22.1
*SGCD*	Sarcoglycan delta	AD, AR	DCM	601411	19q13.32
*SLC25A4*	Solute carrier family 25	AD, AR	DCM	103220	7q21-q22
*TAZ*	Tafazzin	AR, XL	DCM, LVNC	300394	Xq24
*TBX20*	T-Box 20	AD	DCM, LVNC	606061	10q22.3-q23.2
*TCAP*	Titin-Cap	AR	DCM, HCM	604488	3p21
*TMEM43*	Transmembrane protein 43	AD	DCM, ACM	612048	10q23.3
*TMPO*	Thymopoietin	AD	DCM	188380	9q31.2
*TNNC1*	Troponin C type 1	AD	DCM, HCM	191040	17q21.33
*TNNI3*	Troponin I type 3	AD	DCM, RCM, HCM	191044	3p21.1
*TNNT2*	Troponin T type 2	AD	DCM, LVNC, RCM, HCM	191045	17q12
*TOR1AIP1*	Torsin-1a-interacting protein 1	AR	DCM	614512	7q32.1
*TPM1*	Tropomyosin 1	AD	DCM, RCM, HCM	191010	19q13.4
*TRDN*	Triadin	AR	DCM	603283	17q25.3
*TTN*	Titin	AD, AR	DCM, ACM, HCM	188840	5q33-q34
*TXNRD2*	Thioredoxin reductase 2	AD, AR	DCM	606448	8p23.1
*VCL*	Vinculin	AD	DCM, LVNC, HCM	193065	10q25.2

*AD, autosomal dominant; AR autosomal recessive; OMIM, online mendelian inheritance in man.*

Barth syndrome (BTS) is another X-linked cardiac and skeletal myopathy that encompasses abnormal mitochondrial function, short stature, cyclic neutropenia, cardiolipin deficiency, and variable degrees of 3-methylglutaconic aciduria. BTS is caused by mutations in the *TAZ* gene (previously called *G4.5*), which is located in the chromosome Xq28 region and encodes for the Tafazzin protein (Jefferies, 2013). Pathologic gene variants in this gene may result in a wide spectrum of myocardial disease including DCM, HCM, LVNC or a morpho-functional combination of these types. In many cases, affected infants succumb to heart failure, arrhythmias, or sepsis secondary to leukocyte dysfunction (Barth et al., 1983; Towbin, 2010).

Regardless of the mode of inheritance and the pathogenic steps in one or multiple molecular pathways in patients with DCM, progression to heart failure comprises subsequent complex molecular cascades leading to contractile disorganization, metabolic dysregulation, progressive cell death, inflammatory stimulation, remodeling, and heart failure (Bowles et al., 2000; Piran et al., 2012). With disruption of one or multiple proteins or factors implicated in the myocardial mechanics, molecular function and electrical pathways, the ultimate cascade of processes can precipitate the myocardium to become dysfunctional; the “common pathway” in the development of heart failure (Bowles et al., 2000).

Novel sequencing methodologies incorporating panels of genes have enhanced the knowledge of pathogenic genetic variations facilitated the creation of and access to public genomic databases, increasing the probability of finding a pathogenic gene variant. Currently the diagnostic rate of detecting a pathogenic gene variant among patients with non-syndromic DCM range around 40%; this detection rate, however, has not changed after the introduction of conservative criteria to evaluate and define pathogenic variants (Hershberger et al., 2010; van Spaendonck-Zwarts et al., 2013).

## Hypertrophic Cardiomyopathy

**FIGURE 2:**
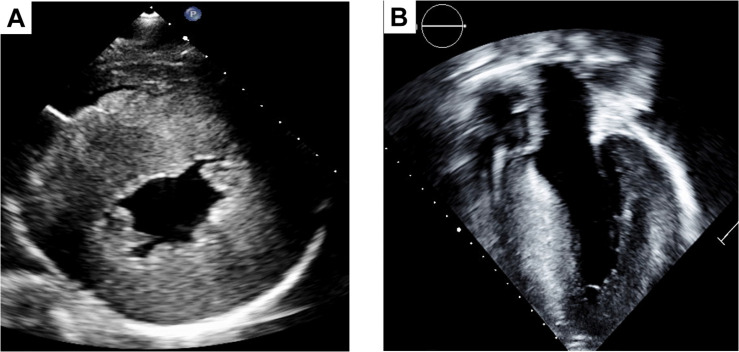
Two dimensional images of HCM in the parasternal short axis **(A)** exhibiting significant concentric hypertrophy and corroborated by the parasternal long axis view **(B)**.

This CM is characterized by left ventricular hypertrophy and histologic evidence of myofiber disarray and interstitial fibrosis (Rowin and Maron, 2013). A cardinal feature of HCM is the presence of asymmetrical hypertrophy, however, a diverse morphology myocardial thickening such as septal, apical and lateral, has been described in association with this disease (Chun et al., 2010) (Figure 2). Mainly, driven by the degree of left ventricular outflow track obstruction, the clinical manifestations present in a wide spectrum ranging from silent, to symptoms of heart failure with preserved ejection fraction, to arrhythmias and sudden death (Brinkley et al., 2020). Diastolic dysfunction can even be detected in individuals with a genetic HCM diagnosis who have normal LV wall thickness, suggesting that diastolic dysfunction is an early feature rather than a consequence of hypertrophy (Rakowski and Carasso, 2009). In the end-stage of the disease, HCM can also present with the “burned-out HCM” phenotype which encompass the syndrome of heart failure with reduced ejection fraction (Brinkley et al., 2020). When echocardiographic data, large-scale epidemiology studies and genetic diagnoses are taken into account, the estimated prevalence of HCM has been raised to 1 case per 200 people in the general population (Maron, 2018). Within the pediatric population, HCM is 10 times more frequent in patients under 1 year of age than in older children, with these younger children more likely to harbor HCM as a syndromic cardiomyopathy (Tables 3, 4) (Colan et al., 2007). HCM typically has an autosomal pattern of inheritance. Although genetic testing alone for the diagnosis of HCM is not recommended, it has a great value in combination of a systematic evaluation for two main reasons. First, the identification of syndromic/genetic diseases known to cause HCM (i.e., Friederichs’s ataxia, Fabry disease, Pompe disease, etc.), and second, the identification of family members of the proband harboring pathogenic gene variants.

**TABLE 3:** List of common genes and patterns of inheritance associated with HCM.

Gene	Protein	Mode of inheritance	Disease association	OMIM#	Locus
*ACTC1*	Actin alpha	AD	HCM, DCM, LVNC, ACM	102540	5q31.1
*ACTN2*	Actinin alpha 2	AD	HCM, DCM	102573	6q22.1
*ALPK3*	Alpha kinase 3	AR	HCM, DCM	617608	1p36.32
*ANKRD1*	Ankyrin repeat domain containing protein 1	AD	HCM, DCM	609599	7q36.1
*BAG3*	Bcl2 associated athanogene 3	AD	HCM, DCM, RCM	603883	14q24.3
*BRAF*	B-Raf proto-oncogene serine/threonine kinase	AD	HCM	164757	12q15
*CAV3*	Caveolin 3	AD	HCM, DCM	601253	12q24.13
*CSRP3*	Cysteine and glycine rich protein 3	AD	HCM, DCM	600824	12p12.1
*FHL1*	Four and a half LIM domains 1	XL	HCM	300163	15q22.31
*FLNC*	Filamin C	AD	HCM, ACM, DMC, RCM	102565	10q22.2
*GAA*	Glucosidase alpha	AR	HCM	606800	19p13.3
*GLA*	Galactosidase alpha	XL	HCM	300644	18q11.2
*HRAS*	HRas proto-oncogene, GTPase	AD	HCM	190020	9q31.1
*JPH2*	Junctophilin 2	AD	HCM	605267	11p11.2
*LAMP2*	Lysosome-associated membrane protein 2	XL	HCM, DCM	309060	3p25.2
*LDB3*	Lim domain binding 3	AD	HCM, DCM, LVNC, ACM	605906	2p22.1
*LMNA*	Lamin A/C	AD, AR	HCM, DCM, LVNC, ACM	150330	1q22
*MAP2K1*	Mitogen activated protein kinase 1	AD	HCM	176872	14q12
*MAP2K2*	Mitogen activated protein kinase 2	AD	HCM	601263	12q24.11
*MYBPC3*	Myosin binding protein C	AD	HCM, DCM, LVNC, RCM	600958	Xq28
*MYH6*	Myosin, heavy chain 6	AD	HCM, DCM	160710	10q25.2
*MYH7*	Myosin, heavy chain 7	AD	HCM, DCM, LVNC, RCM	160760	7p14.2
*MYL2*	Myosin light chain 2	AD	HCM	160781	3p21.3-p21.2
*MYL3*	Myosin light chain 3	AD, AR	HCM, RCM	160790	1q32
*MYLK2*	Myosin light chain kinase 2	AD	HCM	606566	20q13.31
*MYOM1*	Myomesin 1	AD	HCM	603508	18p11.31
*MYOZ2*	Myozenin 2	AD	HCM, DCM, RCM	605602	3p25.1
*MYPN*	Myopalladin	AD	HCM, DCM, RCM	608517	12q23.1
*NEXN*	Nexilin	AD	HCM, DCM	613121	1q22
*NRAS*	Neuroblastoma Ras viral oncogene homolog	AD	HCM	164790	5q31.2
*PDLIM3*	Pdz and Lim domain protein 3	AD	HCM, DCM	605899	1q43
*PLN*	Phospholamban	AD	HCM, DCM, ACM	172405	4q12
*PRKAG2*	Protein kinase Amp-activated non-catalytic gamma-2	AD	HCM	602743	4q26-q27
*PTPN11*	Protein tyrosine phosphatase non-receptor type 11	AD	HCM	176876	10q21.3
*RAF1*	V-Raf-1 murine leukemia viral oncogene homolog 1	AD	HCM	164760	10p12
*RIT1*	Ras-like without Caax 1	AD	HCM	609591	1p31.1
*RYR2*	Ryanodine receptor 2	AD	HCM, ACM	180902	12p11
*SHOC2*	Soc-2 homolog	AD	HCM	602775	5q35.1
*SOS1*	Son of sevenless Drosophila homolog 1	AD	HCM	182530	1p13.2
*TCAP*	Titin-cap	AR	HCM, DCM	604488	3p21
*TNNC1*	Troponin C type 1	AD	HCM, DCM	191040	17q21.33
*NNI3*	Troponin I type 3	AD	HCM, DCM, RCM	191044	3p21.1
*TNNT2*	Troponin T type 2	AD	HCM, DCM, LVNC, RCM	191045	17q12
*TPM1*	Tropomyosin 1	AD	HCM, DCM, RCM	191010	19q13.4
*TTN*	Titin	AD, AR	HCM, DCM, ACM	188840	5q33-q34
*TTR*	Transthyretin	AD	HCM	176300	4q35.1
*VCL*	Vinculin	AD	HCM, DCM, LVNC	193065	10q25.2

*AD, autosomal dominant; AR autosomal recessive; OMIM, online mendelian inheritance in man.*

**TABLE 4:** Typical features of common types of syndromic hypertrophic cardiomyopathy (phenocopies).

Disease	Common pathogenic gene variants and protein product	Patterns of inheritance	Clinical features
Anderson-Fabry disease	*GLA* – α-galactosidase A	XL	Peripheral neuralgia and autonomic dysfunction. Ischemic strokes and arrhythmias. Angiokeratomas, hearing loss. Microalbuminuria and kidney failure. Female carriers are variably affected. The presentation starts during adolescence.
Danon disease	*LAMP2* – lysosome-associated membrane protein 2	XL	Skeletal myopathy in proximal muscle groups. Increased serum creatine kinase. Arrhythmias. Female carriers are variably affected.
Friedreich’s ataxia	*FRDA* – Frataxin	AR	Progressive ataxia of the limbs. Progressive skeletal muscle weakness. Onset varies throughout adulthood.
Kearns–Sayre syndrome	Mitochondrial DNA	Mitochondrial	Pigmentary retinopathy. Progressive external ophthalmoplegia. Onset before 20 years. cardiac conduction defects. Increased CSF protein concentration. Cerebellar ataxia.
Noonan syndrome	*PTPN11* (50% of Noonan cases) – tyrosine phosphatase SHP-2	AD	Prominent forehead, eyes, webbing of neck. Pulmonary valve stenosis, atrial septal defect. Platelet dysfunction and coagulation factors deficiency. Peripheral lymphedema. Pectus excavatum and growth retardation.
Pompe disease	*GAA* – acid alpha-glucosidase	AR	Progressive weakness of proximal muscles Macroglossia, hepatomegaly, feeding and respiratory difficulties, hearing loss. Arrhythmias. Onset varies from birth (classic infantile) through adulthood.

*AD, autosomal dominant; AR, autosomal recessive; XL, X-linked; CSF, cerebrospinal fluid.*

Categorization of HCM includes non-syndromic HCM (without other systemic involvement) and the syndromic form of HCM (in association with inborn errors of metabolism and neuromuscular disorders), also referred as previously mentioned, phenocopies of HCM (Colan, 2010; Popa-Fotea et al., 2019). It has been estimated that 50–60% of patients who have a family member with HCM harbor a pathogenic gene variant (Alfares et al., 2015; Burns et al., 2017).

More than three decades ago, the first chromosome locus (14q11.2-q12) encoding components of the sarcomere (beta-myosin heavy chain) was elucidated as the pathogenic basis for familial HCM (Maass et al., 2002). Since the discovery, more than a thousand mutations in near 30 genes have been implicated in the development of HCM (see Table 3) (Roma-Rodrigues and Fernandes, 2014). Most forms of HCM are inherited in an autosomal dominant transmission, but mitochondrial and autosomal recessive patterns have been also described (Jarcho et al., 1989; Konno et al., 2010; Gersh et al., 2011). Most disease-causing mutations implicated in HCM include pathogenic variants in the *MYH7* gene (encoding beta-myosin heavy chain) and in the *MYBPC3* gene (encoding cardiac myosin-binding protein C). These mutations account individually for 40% of cases; and some other core genes include *TNNT2, TPM1, ACTC1, TNNI3, TTN*, and *MYL2* (Tariq and Ware, 2014). Most of these variants involve missense mutations (resulting in a direct amino acid change) and frameshift-type mutations (insertions or deletions of the number of nucleotides), which alter the properties of the protein involved. Epidemiology studies and the incorporation of genetic panels in clinical practice, have estimated that approximately 70–80% of familial cases have an identified gene variant, whereas fewer mutations (approximately 20%) are identified as infantile HCM (Jarcho et al., 1989; Konno et al., 2010; Gersh et al., 2011; Lipshultz et al., 2019). Genetic defects encoding for a sarcomeric proteins can disrupt the contractile mechanics and the of calcium homeostasis in the sarcomere resulting in a remodeling process by several transcription factors and cardiomyocyte hypertrophy, which eventually results in ischemia, fibrosis, and arrhythmias (Gersh et al., 2011).

### RAS/MAPK Pathway Syndromes

Since the discovery of the first gene (*PTPN11*) associated with Noonan syndrome in 2001, multiple genes (*RAF1, SOS1, KRAS, NRAS, BRAF, MAP2K1, MAP2K2, HRAS*, and *SHOC2*) have been identified in the RAS/mitogen-activated protein kinase pathway. An important molecular corridor managing cell proliferation and differentiation. Thus, dysregulation results in a spectrum of disorders known as “RASopathies” including Noonan and Noonan-like syndromes (Rauen et al., 2010). Children with Noonan-associated syndromes who present HCM and heart failure have a worse risk profile when compared to other children with sarcomeric HCM (Wilkinson et al., 2012).

The management of RASopathies should involve a multidisciplinary team with expertise in the assessment of cardiac structural defects, HCM, and arrhythmias (Table 4).

Congenital metabolic disorders result from absent or abnormal enzymes—or their cofactors—which can lead to accumulation or deficiency of a specific metabolite. Although these disorders exhibit different modes of inheritance, most are transmitted in an autosomal recessive or mitochondrial pattern.

Pompe disease, is a glycogen storage disorder (type II) with autosomal recessive mode of inheritance affecting neuromuscular and myocardial tissues. This disease has broadly classified into the infantile onset (within the first year of life) and the late onset (from childhood to adulthood). The underlying mechanisms include the development of autophagy and the continuous accumulation of glycogen lysosomes secondary to a deficient acid alpha-glucosidase (Hirschhorn and Huie, 1999). The clinical presentation varies in severity and it notably affects skeletal muscle, myocardium and the central nervous system. In the infantile form, musculoskeletal weakness may prevent adequate respiratory mechanics and a normal development. In the myocardium, the build-up of glycogen leads to a progressive phenocopy of hypertrophic cardiomyopathy (Ruiz-Guerrero and Barriales-Villa, 2018). Electrocardiography (ECG) reveals a short PR interval, a manifest delta wave, and giant QRS complexes in all leads, suggesting biventricular hypertrophy. Chronic troponinemia may be observed based on our clinical experience. Other findings include elevated creatine kinase, lactate dehydrogenase, and aspartate aminotransferase. The juvenile and adult forms present with variable age of onset. Enzyme replacement therapy has shown to decrease ventricular hypertrophy, LV outflow tract obstruction, and normalization of electrocardiographic findings (Levine et al., 2008).

Danon disease, is a glycogen storage disorder (type IIb) with X-linked mode of inheritance incorporating with skeletal muscle weakness and intellectual disabilities. Danon disease involves a genetic defect in the *LAMP2* gene located at chromosome Xq24, which encodes the lysosome-associated membrane protein. This lysosomal storage disease mimics the phenotypic features of the sarcomeric-gene-associated HCM. However, in Danon disease the presence of ventricular preexcitation is more frequently encountered than in those individuals with sarcomeric HCM (Arad et al., 2005; Rowland et al., 2016). The cardiac degeneration is usually appreciated clinically by the presence of HCM during childhood or adolescence with subsequent transition to a DCM phenotype with progressive heart failure (Maron et al., 2009). Female carriers have also been described in this disorder and are attributed to unfavorable X chromosome inactivation (lyonization) causing the development of symptoms around the fourth decade of life (Toib et al., 2010).

Fabry disease is an X-linked lysosomal disease secondary to a deficient hydrolase alpha-galactosidase A (Brady et al., 1967). Most cases are familial and few originate from *de novo* mutations (Garman and Garboczi, 2004). Patients with Fabry disease may present with a multisystem involvement including neurologic (paresthesia and pain crises), dermatologic (angiokeratomas and telangiectasias), ophthalmologic (corneal dystrophy), renal (proteinuria and renal insufficiency), and cardiac manifestations by the second to fifth decades of life (Mehta et al., 2004). Cardiac disease is relatively common in Fabry disease. Patients may develop a HCM phenocopy (similar to that seen in sarcomeric HCM), arrhythmias, and valvar abnormalities. Enzyme replacement therapy has shown attenuation of cardiovascular compromise (Perrot et al., 2002; O’Mahony and Elliott, 2010).

Friedreich’s ataxia is another disease expressed as a phenocopy of HCM affecting approximately 1 in 20,000 – 750,000 individuals (Burk, 2017). From the genetic standpoint, the inheritance mode is autosomal recessive, and it is characterized by trinucleotide repeat expansion of a normal codon affecting the protein frataxin, a mitochondrial protein implicated in iron homeostasis required for the mitochondrial electron transport chain. The trinucleotide repeat expansion causes a reduction in the frataxin levels. Thus, the bigger the expansion, the earlier the age of onset and the severity of the disease (Patel and Isaya, 2001; Burk, 2017). The major clinical manifestations of Friedreich’s ataxia appear during childhood and adolescence, and include progressive neurologic dysfunction (gait ataxia, optic atrophy, loss of position and vibration sense), diabetes mellitus, and myocardial involvement. The cardiac phenotype is manifested by arrhythmias and HCM. Heart failure remains the leading cause of death in this population (Kipps et al., 2009; Peverill, 2012; Weidemann et al., 2012).

## Left Ventricular Non-Compaction Cardiomyopathy

**FIGURE 3:**
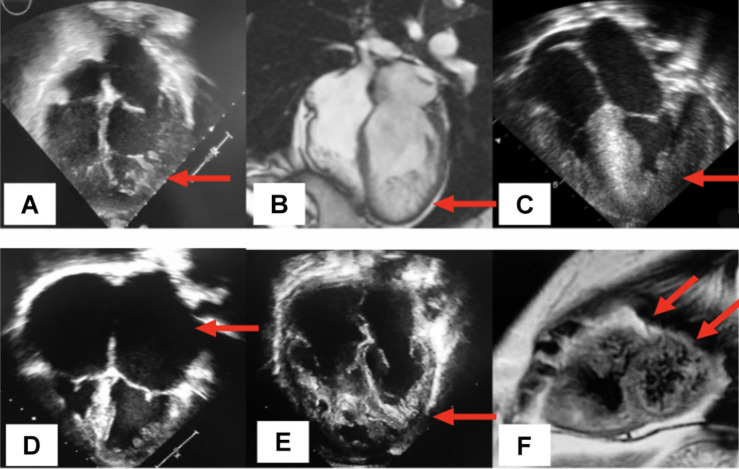
Distinct phenotypes of non-compaction cardiomyopathy. **(A)** Echocardiographic 4-chamber view displays the isolated type of LVNC illustrated by the cardinal feature of left ventricular trabeculations (arrow) with normal anatomy and function; **(B)** from a cardiac magnetic resonance imaging (CMRi), a 4-chamber view displays the dilated sun-type of LVNC, denoting the enlargement of the LV and the presence of inferolateral trabeculations (arrow); **(C)** echocardiographic 4-chamber view shows the hypertrophic type of LVNC represented by asymmetric hypertrophy of the interventricular septum and the presence of lateral LV trabeculations (arrow); **(D)** display of the restrictive type of LVNC, significant bilateral atrial enlargement (arrows) and the presence of left ventricular trabeculations; **(E)** biventricular hypertrabeculations (arrows); **(F)** cMRI in a short axis view displays a mixed LVNC phenotype represented by dilated and dysfunctional ventricles in a patient with ventricular arrhythmias and biventricular trabeculations secondary to a pathogenic variant in the *PRDM16* gene (arrows).

LVNC is characterized by multiple prominent ventricular trabeculations, intertrabecular deep recesses, and myocardium with two distinct layers: a non-compacted subendocardial layer and a thin compacted epicardial layer (Towbin, 2010). LVNC is presumably caused by abnormal *in utero* myocardial compaction, a final stage of myocardial morphogenesis (Zhang et al., 2013; Liu et al., 2018). However, this hypothesis does not explain why LVNC manifests in a wide variety of sub phenotypes (Table 5) with a heterogeneous clinical course. LVNC also shows increased incidence in certain physiologic states, such as pregnancy (Gati et al., 2014) and vigorous physical activity (Gati et al., 2013; de la Chica et al., 2020). It is also seen in association with other conditions such as in patients with congenital heart disease (CHD), neuromuscular disorders, mitochondrial disease and metabolic derangements (Finsterer et al., 2017; Martinez et al., 2017; Ross et al., 2020; Vershinina et al., 2020). Since the initial description in 1926, LVNC has been coined with various names, including spongiform myocardium, fetal or primordial myocardium, hypertrabeculation syndrome, and non-compaction cardiomyopathy (Maron et al., 2006; Engberding et al., 2007). Currently, this cardiomyopathy is categorized as a primary genetic cardiomyopathy by the American Heart Association and as a primary morphofunctional cardiomyopathy by the MOGE(s) nosology (Maron et al., 2006; Elliott et al., 2008; Arbustini et al., 2014). Because the genesis of LVNC is incompletely understood, diagnosis typically relies on non-invasive imaging, Figure 3. Since the 1990s, many diagnostic criteria have been proposed based on morphological characteristics of the myocardium seen by echocardiography and cardiac magnetic resonance. Without analytic and universally accepted criteria, the prevalence of LVNC is difficult to estimate, but the approximate range is estimated in the 0.014–1.3% range of patients undergoing echocardiography (Pignatelli et al., 2003; Stanton et al., 2009). However, among patients with heart failure and patients with severe CHD, the prevalence has been reported at around 4 and 7.5%, respectively (Kovacevic-Preradovic et al., 2009; Martinez et al., 2017). In this review, we provide an informative categorization of 9 distinct subphenotypes of LVNC (Towbin and Beasley, 2020) (Table 5). The overall clinical manifestation is heterogenous ranging from asymptomatic to a severe course accompanied by heart failure requiring heart transplant, arrhythmias, sudden cardiac death, and thromboembolic phenomena (Lee et al., 2017). Familial cases are well-documented, and autosomal dominant transmission is currently the most recognized inheritance pattern (with variable penetrance and phenotypic heterogeneity). Other heritable modes of transmission include X-linked, autosomal recessive, and mitochondrial (Roma-Rodrigues and Fernandes, 2014). In pediatric and adult cohorts, the rate of finding a pathogenic gene variant in patients manifesting LVNC ranges from 17 to 41% depending on patient selection and the cardiomyopathy gene panels (Sedaghat-Hamedani et al., 2017; van Waning et al., 2019). One of the earliest locus associations with LVNC was described in 1997 in the gene *G4.5/TAZ* located at chromosome Xq28 (Lee et al., 2017). Since then, multiple pathologic gene variants have been described as potential causes of LVNC. Genes encoding sarcomeric and cytoskeletal proteins (*TTN, ACTN2, RBM20, LMNA, DES*, DYS, *DTNA, LDB3, MYH7, MYBPC3*, and *ACTC1*) as well as genes associated with cardiac morphogenesis (*FKBP12, MIB1, Tbx20, Nkx2-5, Smad7, NF-ATc*, and *Jarid2*), ion channels (*SCN5A*, *HCN4*, and *RYR2*), and mitochondria (*NNT, TAZ*) have been implicated in the development of LVNC (Bagnall et al., 2014; Hastings et al., 2016; van Waning et al., 2018, 2019). Along with sarcomere-encoding and cytoskeleton-encoding genes, pathogenic variants in a variety of genes, including *SCN5A*, *LMNA*, *RBM20*, *TTN*, and *DES*, have been associated with LVNC and rhythm disturbance (Finsterer and Stollberger, 2008; Shan et al., 2008) (Table 6). Mutations in the mitochondrial genome and chromosomal abnormalities have also been linked to LVNC, including 1p36 deletion (encompassing the PRDM16 gene), 7p14.3p14.1 deletion, 22q11.2 deletion, distal 22q11.2, and other chromosomal trisomies (Towbin, 2010; Tariq and Ware, 2014; Dong et al., 2017; Miller et al., 2017; Miszalski-Jamka et al., 2017; van Waning et al., 2018).

**TABLE 5:** Subphenotypes in the clinical spectrum of left ventricular non-compaction.

Subtype	Characteristics
Isolated form	Abnormal trabeculations with normal cardiac dimension and function
Arrhythmogenic form	Isolated form with supraventricular or ventricular tachycardias
Dilated form	Dilated and hypertrabeculated LV with systolic dysfunction
Hypertrophic form	Concentric and asymmetric hypertrophy with LV trabeculations
Restrictive form	Bilateral atrial enlargement, diastolic dysfunction, and LV trabeculations
Mixed form	A mix of characteristics from hypertrophic, dilated, and restrictive phenotypes
Biventricular form	Trabeculation in the right and left ventricles
Right ventricular form	Trabeculation in the right ventricle
Congenital heart disease form	LV trabeculations associated with concomitant congenital heart disease

**TABLE 6:** List of common genes and patterns of inheritance associated with LVNC.

Gene	Protein	Mode of inheritance	Disease association	OMIM#	Locus
*ACTC1*	Actin, alpha, cardiac muscle	AD	LVNC, ACM, HCM, DCM	102540	5q31.1
*CASQ2*	Calsequestrin 2	AR, AD	LVNC	114251	6q22.31
*DTNA*	Dystrobrevin, alpha	AD	LVNC	601239	2q31
*HCN4*	Hyperpolarization-Activated. Cyclic nucleotide-gated. Potassium Channel 4	AD	LVNC	605206	18q12.1
*LDB3*	Lim domain-binding 3	AD	LVNC, ACM, HCM, DCM	605906	2p22.1
*LMNA*	Lamin A/C	AD, AR	LVNC, ACM, HCM, DCM	150330	1q22
*MIB1*	E3 ubiquitin protein ligase 1	AD	LVNC	608677	22q11.21
*MYBPC3*	Myosin-binding protein C, cardiac	AD	LVNC, RCM, HCM, DCM	600958	Xq28
*MYH7*	Myosin, heavy chain 7, cardiac muscle, beta	AD	LVNC, RCM, HCM, DCM	160760	7p14.2
*PRDM16*	Pr domain-containing protein 16	AD	LVNC, DCM	605557	6q21
*TAZ*	Tafazzin	AR, XL	LVNC, DCM	300394	Xq24
*TBX20*	T-box 20	AD	LVNC, DCM	606061	10q22.3-q23.2
*TNNT2*	Troponin T type 2 (cardiac)	AD	LVNC, RCM, HCM, DCM	191045	17q12
*VCL*	Vinculin	AD	LVNC, HCM, DCM	193065	10q25.2

*AD, autosomal dominant; AR autosomal recessive; OMIM, online mendelian inheritance in man.*

## Arrhythmogenic Cardiomyopathy (ACM)

Arrhythmogenic cardiomyopathy is myocardial disorder unrelated to coronary artery disease, hypertension, or valvular heart disease. ACM was formerly known as arrhythmogenic right ventricular dysplasia/cardiomyopathy (ARVD/ARVC). The prevalence has been estimated at around 1 in 1,000 – 5,000 people (Peters et al., 2004). The clinical diagnosis may be supported by evidence of conduction disease, supraventricular arrhythmias, and/or ventricular arrhythmias originating from any cardiac structure. ECG abnormalities include right bundle branch block pattern, epsilon wave (a low-amplitude deflection between the end of the QRS and the onset of the T wave in the precordial leads V1 – V3), and T wave inversion in the precordial leads V1 – V4. Classically, the RV is dilated and contains fibro-fatty infiltration of the myocardium. The left ventricle is overtly affected less frequently. Thus, ACM should be categorized into right dominance, left dominance, or biventricular involvement.

Notably, ACM clinically overlaps with other CM types, particularly DCM. However, ACM is distinct because it is marked by arrhythmia at presentation with or without biventricular dilatation and/or impaired systolic function (Towbin et al., 2019). This heritable disorder is primarily transmitted in an autosomal dominant pattern, but autosomal recessive heritability has been reported in families with cardiocutaneous disease (Greece, Italy, India, Ecuador, Israel, and Turkey) (Protonotarios and Tsatsopoulou, 2004). The most notable autosomal recessive diseases include Naxos disease (a homozygous pathogenic variant in the gene encoding the protein plakoglobin, characterized by ACM, non-epidermolytic palmoplantar keratoderma, and wooly hair) and Carvajal syndrome (caused by a homozygous pathogenic gene variant that truncates the DSP protein) (Protonotarios and Tsatsopoulou, 2004; Marcus et al., 2010). Studies have suggested that up to 50% of the cases are familial (Hamid et al., 2002). Pathogenic gene variants within the desmosomal proteins are the main cause of the “classic” ACM (Bauce et al., 2010). Pathogenic gene variants in desmosomal proteins have significant implications in the development of ACM and account for up to 60% of affected patients (den Haan et al., 2009; Delmar and McKenna, 2010). Overall, the most commonly mutated gene is plakophilin, which accounted for 46–61% of patients from two different registries (Groeneweg et al., 2015). To date, about 18 causative genes involved in ACM have been identified (Hamid et al., 2002; Towbin et al., 2019) (Table 7).

**TABLE 7:** List of common genes and patterns of inheritance associated with ACM.

Gene	Protein	Mode of inheritance	Disease association	OMIM#	Locus
*ACTC1*	Actin, alpha, cardiac muscle	AD	ACM, HCM, DCM, LVNC	102540	5q31.1
*ARVD3*	Arrhythmogenic right ventricular dysplasia, familial, 3	AD	ACM	602086	12p12.1
*ARVD4*	Arrhythmogenic right ventricular dysplasia, familial, 4	AD	ACM	602087	15q14
*ARVD6*	Arrhythmogenic right ventricular dysplasia, familial, 6	AD	ACM	604401	1q42-q43
*CTNNA3*	Catenin alpha 3	AD	ACM	607667	7q21.2
*DES*	Desmin	AD, AR	ACM, RCM, DCM	125660	17q21
*DSC2*	Desmocollin 2	AD, AR	ACM, DCM	600271	Xq22
*DSG2*	Desmoglein 2	AD	ACM, DCM	125671	15q24.1
*DSP*	Desmoplakin	AD, AR	ACM, DCM	125485	11p15.5
*FLNC*	Filamin C	AD	ACM, DMC, RCM, HCM	102565	10q22.2
*JUP*	Junction plakoglobin	AD, AR	ACM	173325	2p13.1
*LDB3*	Lim domain-binding 3	AD	ACM, HCM, DCM, LVNC	605906	2p22.1
*LMNA*	Lamin A/C	AD, AR	ACM, HCM, DCM, LVNC	150330	1q22
*PKP2*	Plakophilin 2	AD	ACM, DCM	602861	11p15.4
*PLN*	Phospholamban	AD	ACM, HCM, DCM	172405	4q12
*RYR2*	Ryanodine receptor 2 (cardiac)	AD	ACM, HCM	180902	12p11
*SCN5A*	Sodium channel, voltage-gated, type V, alpha subunit	AD	ACM, DCM	600163	20q13.12
*TGFB3*	Transforming growth factor beta 3	AD	ACM	190230	
*TMEM43*	Transmembrane protein 43	AD	ACM	612048	10q23.3
*TTN*	Titin	AD, AR	ACM, HCM, DCM	188840	5q33-q34

*AD, autosomal dominant; AR autosomal recessive; OMIM, online mendelian inheritance in man.*

## Restrictive Cardiomyopathy (RCM)

**FIGURE 4:**
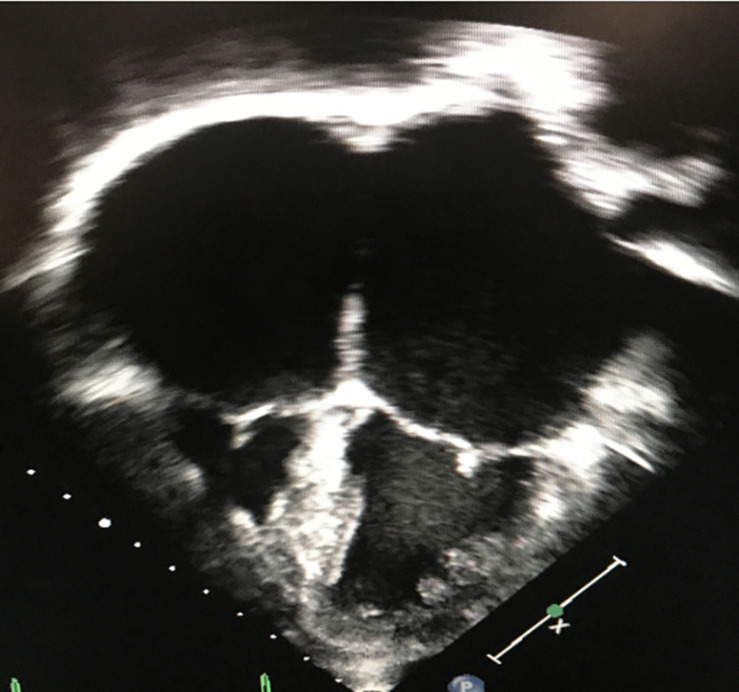
Two-dimensional, apical 4-chamber echocardiographic image depicting small, restrictive ventricles and significant biatrial enlargement in a patient with restrictive cardiomyopathy.

Restrictive cardiomyopathy is characterized by abnormal diastology with typically preserved ejection fraction, relatively normal ventricular dimensions and the presence of atrial enlargement. The underlying hemodynamic consequence includes elevated ventricular filling pressures and a restrictive physiology (Maron et al., 2006; Denfield and Webber, 2010) (Figure 4).

The clinical presentation is heterogenous and it relies on the degree of the inability to accommodate adequate cardiac input into the ventricles due to abnormal myocardial pliability or diastolic relaxation. Clinical symptoms may include exercise intolerance, dyspnea, volume overload, tachyarrhythmias, syncope, or sudden cardiac death. The hallmark of non-invasive imaging is atrial or bi-atrial enlargement. Normal or mild concentric hypertrophy with normal or reduced ventricular cavity can also be seen. Familial cases contribute in up to 30% of cases in RCM, and it usually exhibits autosomal dominant, autosomal recessive, X-linked, and mitochondrial transmitted modes of inheritance (Kaski et al., 2008). RCM can be classified based on the underlying process: non-infiltrative or primary; infiltrative (or systemic); associated with storage diseases (or syndromic); idiopathic; or in combination with other morpho functional types, such as DCM, HCM, and LVNC (Denfield and Webber, 2010). Primary myocardial disease or non-infiltrative types have been attributed to pathogenic gene variants in sarcomeric genes, such as *TNNI3* (most common), *TNNT2, MYH7, ACTC1, TPM1, MYL3*, and *MYL2*, (Table 8) (Caleshu et al., 2011; Towbin, 2014). Scleroderma and other pathologies in the connective tissues are also considered causes of genetically triggered RCM (Worthley et al., 2001; Nguyen et al., 2006). Infiltrative causes of RCM are mainly represented by amyloidosis (the most common type), sarcoidosis and hemochromatosis. The former, encompass a group of metabolic derangements characterized by deposition of insoluble fibrillar protein-like metabolites altering organ function and structure (including the myocardium). There are approximately 20 different proteins associated with cardiac amyloidosis, with TRR, AL, ATTRm, ATTRwt, and ApoA-I representing the most common types (Rapezzi et al., 2009; Brown et al., 2021). Sarcoidosis is a multisystemic granulomatous process affecting the lungs, skin and myocardium. The incidence varies with geography and ethnic groups, for instance, it has been described to affect black > white Americans, and female > male patients. The strongest genetic associations are embedded in the major histocompatibility complex and *BTNL2* gene (Spagnolo et al., 2007). Lysosomal storage disorders are characterized by abnormal lysosomal metabolism leading to the accumulation of various glycosaminoglycans, glycoproteins, or glycolipids within lysosomes of various tissues, including the myocardium. Gaucher disease and Fabry disease (two of the most common lysosomal disorders) may manifest as CM (HCM or RCM), valvular disease, coronary artery disease, and/or aortic enlargement (Lindor and Karnes, 1995). Hemochromatosis is a disorder typically characterized by increased intestinal iron absorption leading to iron overload and systemic deposition. This disease has been associated with low-penetrance autosomal dominance of pathogenic variants identified in the *HFE* gene (Pietrangelo, 2010, 2015). Glycogen storage disorders such Hurler disease and Hunter disease are characterized by the existence of deficient enzymes necessary for the breakdown of glycosaminoglycans. When these disorders manifest, cardiac involvement incorporate impaired ventricular filling, endocardial fibroelastosis, and cardiac valvulopathy (stenosis and/or insufficiency) (Brown et al., 2021).

**TABLE 8:** List of common genes and patterns of inheritance associated with RCM.

Gene	Protein	Mode of inheritance	Disease association	OMIM#	Locus
*BAG3*	Bcl2-associated athanogene 3	AD	LVNC, HCM, DCM	603883	14q24.3
*DES*	Desmin	AD, AR	RCM, DCM, ACM	125660	17q21
*FLNC*	Filamin C	AD	RCM, HCM, ACM, LVNC	102565	10q22.2
*MYBPC3*	Myosin-binding protein C, cardiac	AD	RCM, HCM, DCM, LVNC	600958	Xq28
*MYH7*	Myosin, heavy chain 7, cardiac muscle, beta	AD	RCM, HCM, DCM, LVNC	160760	7p14.2
*MYL3*	Myosin, light chain 3, alkali, ventricular, skeletal, slow	AD, AR	RCM, HCM	160790	1q32
*MYOZ2*	Myozenin 2	AD	RCM, HCM, DCM	605602	3p25.1
*MYPN*	Myopalladin	AD	RCM, HCM, DCM	608517	12q23.1
*TNNI3*	Troponin I type 3 (cardiac)	AD	RCM, HCM, DCM	191044	3p21.1
*TNNT2*	Troponin T type 2 (cardiac)	AD	RCM, HCM, DCM, LVNC	191045	17q12
*TPM1*	Tropomyosin 1 (alpha)	AD	RCM, HCM, DCM	191010	19q13.4

*AD, autosomal dominant; AR autosomal recessive; OMIM, online mendelian inheritance in man.*

## Clinical Genetic Discussion

A methodical approach to the genetic diagnosis of heritable cardiomyopathies should be initiated by obtaining a detailed history of present illness, a complete family history incorporating a three-generation pedigree, and a physical examination with attention to syndromic cardiovascular disease (Musunuru et al., 2020). Care should be taken when interpreting the family pedigrees, as they may not represent updated information in followed-up visits, variable expressivity in patients, bilateral genotypic predisposition and the role of epigenetics (Hershberger et al., 2013).

The review of cardiovascular imaging, electrocardiographic data, and the incorporation of functional cardiovascular assessments provides informative data to stratify disease, identify morbidity and to guide clinical and individual management of myocardial diseases (Table 9). In “classic” phenotypic characteristics, pattern-recognition of syndromic disease may guide clinicians to selecting the best targeted medical management. The selection and initiation of genetic testing should be guided by the aforementioned information. Current guidelines recommend genetic testing in children and adult patients harboring heritable cardiomyopathies (Ingles et al., 2011; Hershberger et al., 2018b).

**TABLE 9:** Initial workup of heritable cardiomyopathies.

Physical examination with special attention to syndromic cardiovascular disease
Family history with generation of at least a 3-generation pedigree
Review of imaging and electrocardiographic findings
Diagnostic and screening recommendations for metabolic cardiomyopathies based on the presentation
Consideration of genetic testing
Genetic counseling

When genetic results are interpreted, we recommend bearing in mind the following concepts. Penetrance, which describes the phenomenon by whether a phenotype can be observed on an individual harboring pathogenic genotype. For instance, most autosomal dominant cardiomyopathies (associated with sarcomeric mutations) are characterized by incomplete penetrance by adulthood. Whereas autosomal recessive cardiomyopathies are associated with complete penetrance before adulthood (Charron et al., 2010). Variable expressivity, this term entitles the description of variable phenotypic manifestations among carriers of a particular pathogenic gene variant (Charron, 2006; Balistreri et al., 2019).

Genetic testing should be perceived as a complementary study in the diagnosis of cardiomyopathy.

The major critical aspect of integrating genetic testing to study the first affected individual (proband), is to refine diagnoses in syndromic and non-syndromic cardiomyopathies, as early identification of genetically predisposed individuals allows for disease stratification, predicts morbidity, individualizes medical management, and in some cases, it provides genotype-specific therapy. Similarly, genetic testing should be routinely incorporated to screen family members of the proband harboring the risk of hosting similar pathogenic gene variants. This approach is encouraged by the existence of high prevalence of heritability in first-degree relatives.

### Future Directions

The field of cardiomyopathy and genetics should continue to sum efforts to accomplish research goals and improve patient healthcare. Precision medicine from genomics and personal health history should be integrated in three areas in particular: (1) to create gene-based therapies for individual patients, (2) to enhance preventive medicine to transform health care systems from illness to prevention and wellness, and (3) by better understanding disease modifiers and epigenetics in cardiovascular disease. For those patients who harbor CM as a chronic illness, we found that the quality of life and the quality of outcomes for these survivors should be continuously assessed by healthcare providers, guardians and family members. Academic and investigational projects driving improvements in outcomes of cardiomyopathy patients are important since determinants of health wellbeing and disease exist in our communities. This topic is of importance, as there exist a tremendous relatively untapped set of opportunities to create synergy between clinicians, policy makers, advocacy groups and funding institutions.

The domains of precision medicine and artificial intelligence have brought rapid advances and alternatives in research by allowing one to manage big data from electronic medical records and collaborative databases. As a result, we have witnessed the development of molecular compounds (myosin inhibitors, calcium sensitizers, myosin activators, etc.) in the management of various types of cardiomyopathies. These methodologies have also contributed to the utilization of genotype phenotype coupling, pharmaco-genomic profiles, and individualization of therapies based on algorithms supporting the use of predictive analytics. Cardiovascular genetics is currently leading a transition from – making the diagnosis prior to applying gene testing to requesting diagnostic genome testing for an early diagnosis to avoid unnecessary harm – perhaps a natural extension for what it pertains to the cardiomyopathy genes.

## Conclusion

In recent years, the number of individuals diagnosed with hereditary CMs has grown, likely due to increased awareness, advances in imaging modalities, and a better understanding of phenotypic associations and the molecular genesis of these diseases.

In our experience, genetic testing has been most beneficial in CMs associated with other cardiac features, such as LV dysfunction, LV hypertrophy, arrhythmias, and the co-occurrence of other cardiac and non-cardiac syndromic features.

The central principle supporting the incorporation of genetic testing in cardiovascular medicine relies on the allowance of practitioners to recognize at-risk family members and the advantageous strategy to delineate disease, provide adequate surveillance and tailor individual therapeutic options.

Implementation of precision medicine in the evaluation of heritable CMs likely ensures a dramatic improvement in patient outcomes and shifts the current paradigm from a disease-treatment to an early-diagnostic/preventive healthcare system.

## Author Contributions

HM took the lead in writing the manuscript. All authors provided critical feedback and helped shape the manuscript. JLJ conceived the original idea of the project.

## Conflict of Interest

The authors declare that the research was conducted in the absence of any commercial or financial relationships that could be construed as a potential conflict of interest.
